# Author Correction: Infralimbic cortex is required for learning alternatives to prelimbic promoted associations through reciprocal connectivity

**DOI:** 10.1038/s41467-019-11205-w

**Published:** 2019-07-12

**Authors:** Arghya Mukherjee, Pico Caroni

**Affiliations:** Friedrich Miescher Institut, Basel, CH-4058 Switzerland

**Keywords:** Decision, Consolidation, Extinction

Correction to: *Nature Communications* 10.1038/s41467-018-05318-x, published online 13 July 2018.

The original version of this Article contained an error in the Acknowledgements, which incorrectly omitted from the end the following: ‘This project has received funding from the European Research Council (ERC) under the European Union’s Horizon 2020 research and innovation programme (MemoryDynamics - grant agreement No 694086).’ This has been corrected in both the PDF and HTML versions of the Article.

