# Supplementary material for: Infralimbic cortex is required for learning alternatives to prelimbic promoted associations through reciprocal connectivity
Source: Nat Commun. 2018 Jul 13;9:2727. doi: 10.1038/s41467-018-05318-x (PMC6045592; doi:10.1038/s41467-018-05318-x)
Supplement: Supplementary file 1 — Supplementary Information [file 41467_2018_5318_MOESM1_ESM.pdf]

INFRALIMBIC CORTEX REQUIRED TO LEARN ALTERNATIVES TO PRELIMBIC PROMOTED ASSOCIATIONS THROUGH RECIPROCAL CONNECTIVITY

Mukherjee and Caroni

Supplementary Notes

From IL-dependent to IL- and PreL-dependent extinction learning

Studies of extinction after traumatic experiences in humans have highlighted how extinction protocols are not always effective, and that in some cases extinction can be difficult or even not possible to achieve. Accordingly, we wondered whether under circumstances in which extinction might be difficult to achieve, extinction learning still specifically depends on activity in IL, and on IL->PreL connectivity. Towards this end, we asked whether a standard extinction protocol in mice might fail when fear learning is reinforced on consecutive days. Indeed, fear behavior produced by two tFC protocols delivered on consecutive days resisted extinction (**Suppl. Fig. 6**). In a further experimental protocol that resisted extinction learning, we found that P60 mice that underwent an extinction protocol while IL was silenced (no extinction learning, see **Fig. 4e**), also failed to exhibit extinction on the subsequent day (now without IL silencing; **Suppl. Fig. 7**).

Since silencing PreL slightly accelerated extinction (**Fig. 4e**), we wondered whether silencing PreL might be sufficient to produce detectable extinction learning in these extinction-resistant paradigms. Indeed, silencing PreL during the extinction protocol (PV neuron activation; PSEM delivery 20min before) produced robust extinction learning in mice that had undergone two tFC protocols, or in which extinction learning had been previously prevented by IL silencing (**Suppl. Figs. 6, 7**). Notably, silencing of both, PreL and IL during the extinction protocol again prevented extinction learning, indicating that extinction in the absence of active PreL still depended on active IL (**Suppl. Figs. 6, 7**). In the following, we will use the term Ext1 (as opposed to extinction) to designate such PreL-prevented and IL-dependent extinction learning.

To investigate whether and how extinction learning is still possible under such more challenging conditions, we next searched for behavioral protocols that would produce extinction learning without need for PreL silencing in these extinction-resistant settings. A total of 21 (instead of 6) consecutive tone presentations in the absence of foot shocks (21CS/-) failed to produce detectable extinction learning in extinction-resistant mice (**Suppl. Fig. 7**). Likewise, three blocks of 6CS/- delivered on the same day also failed to produce extinction learning (**Suppl. Fig. 7**). In extinction protocols involving consecutive days, a second block of 6CS/- delivered on a subsequent day (Ext2) led to somewhat reduced freezing values (50-60% instead of 60-70% of the time), but no detectable extinction learning (**Suppl. Figs. 6, 7**). Notably, however, a third block of 6CS/- on a subsequent day (Ext3) now produced robust extinction in the two extinction-resistant models (**Suppl. Figs. 6, 7**). As for conventional extinction learning (one 6CS/- session), freezing to tone had returned 10d after the Ext1-3 extinction protocol, but extinction was now readily achieved in one 6CS/- session, providing evidence for savings of Ext1-3 extinction learning (**Suppl. Fig. 7**).

We then investigated requirements for activity in IL and PreL during Ext1-3 extinction learning. Silencing IL during Ext1, or Ext2 learning suppressed detectable extinction learning upon Ext3, indicating that functional IL at both Ext1 and Ext2 was a prerequisite for extinction learning at Ext3 (**Suppl. Fig. 6**). Notably, and in stark contrast to Ext1 and Ext2, extinction learning at Ext3 depended on activity in both IL and PreL (**Suppl. Fig. 6**). Ext3 extinction learning (PreL-dependent) therefore differs qualitatively from conventional (6CS/-) extinction learning (PreL- independent). Our results suggest that Ext1-3 learning consists of IL-dependent learning sessions (Ext1, Ext2), followed by IL- and PreL-dependent extinction learning (Ext3) (**Suppl. Fig. 6**). The striking dependence on first IL, and then on IL and PreL for behaviorally detectable extinction learning suggests that Ext1-3 extinction learning might represent IL-dependent alternation (away from CS predicts US), followed by PreL-supported learning of the new association (CS is safe) in a way comparable to EDS learning.

Next, we determined whether, like in conventional extinction, activity in IL->PreL projection neurons was specifically required during processes in Ext1-3 extinction learning that depend on IL. Indeed, PreL-prevented, IL-dependent Ext1 learning was suppressed when IL->PreL connectivity was inhibited during Ext1 learning, but not when PreL->IL connectivity was inhibited during Ext1 learning (**Suppl. Fig. 6**). Furthermore, inhibiting IL->PreL connectivity during Ext2 or Ext3 learning suppressed detectable extinction learning at Ext3, whereas inhibiting PreL->IL connectivity during Ext2 or Ext3 learning did not prevent Ext3 learning (**Suppl. Fig. 6**). Taken together, these results further support the notion that

activity in IL->PreL (but not PreL->IL) projection neurons is specifically required during IL-dependent alternative learning.

Supplementary Figures and their Legends

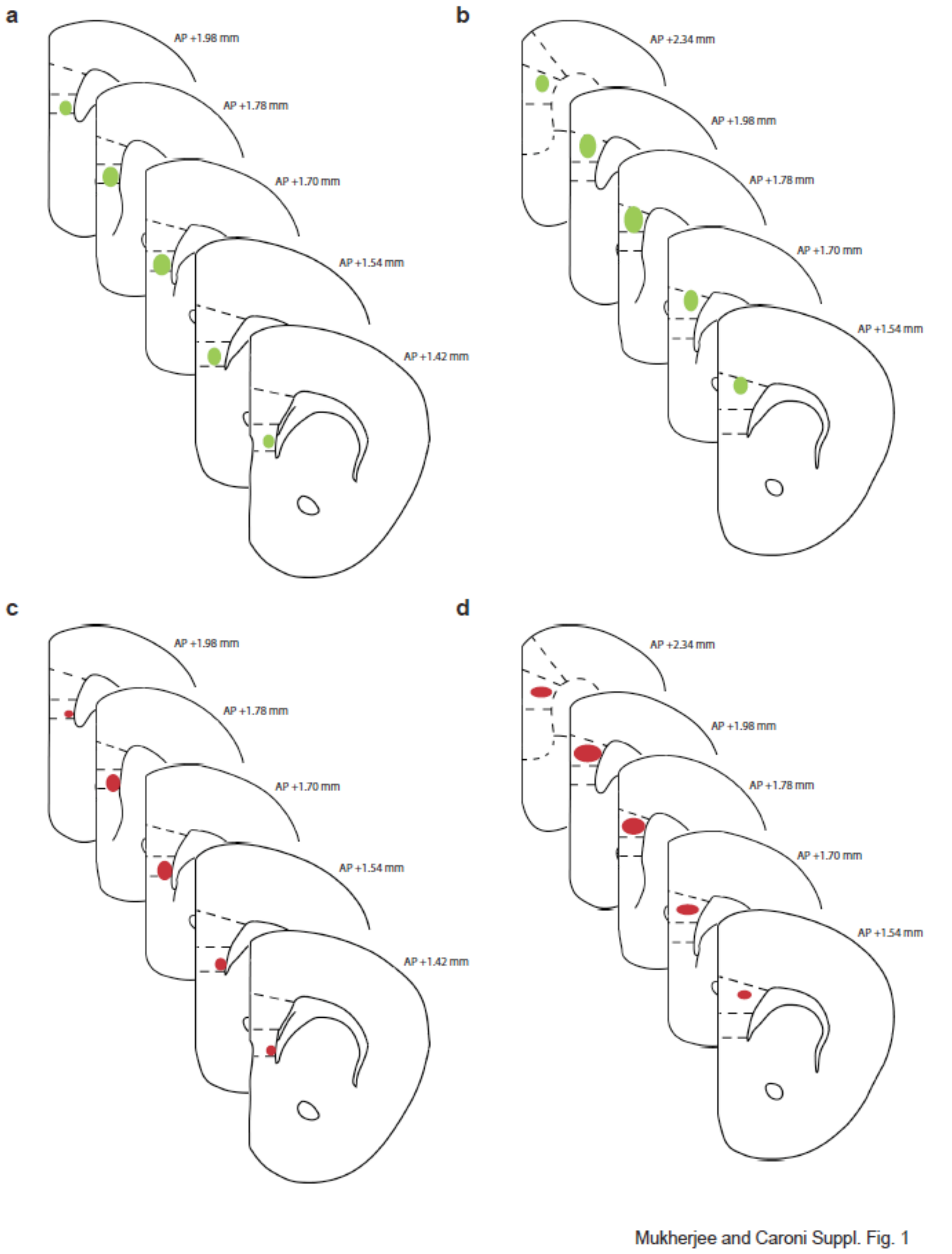

Supplementary Figure 1. **Local viral targeting of PreL or IL neurons for silencing and connectivity experiments.** Representative examples of virus spread range (**a, b**) and of retrograde projection neuron targeting (**c, d**).
a, b: Schematic representations of virus labeling (green, Bungarotoxin488 signal) in IL (**a**) or PreL (**b**) in PV-Cre mice. Such mice were used for IL or PreL silencing experiments.
c, d: Schematic representations of retrograde projection neuron labeling (red, mCherry signal) in IL->PreL (**c**) and PreL->IL (**d**) targeting experiments. Such mice were used for the analysis and manipulation of IL->PreL and PreL->IL projection neurons.

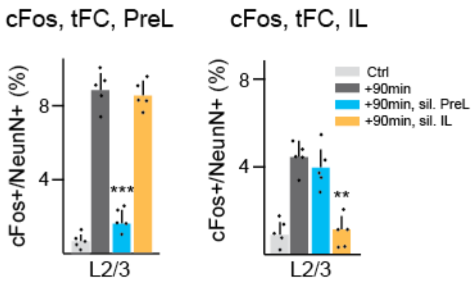

Mukherjee and Caroni Suppl. Fig. 2

Supplementary Figure 2. **Local silencing of PreL or IL: impact on cFos expression.** Silencing through pharmacogenetic PV-neuron activation in PV-Cre mice during acquisition of trace fear conditioning (tFC). cFos+ neuron contents 90 min after acquisition in PreL or IL. Note loss of cFos+ neurons specifically in PreL upon PreL silencing (blue; n=5 each; F (3, 16) = 36.84, ***P), and in IL upon IL silencing (orange; n=5 each; F (3, 16) = 28.73, ***P). One-way ANOVA followed by Dunnet's *post hoc*; P<0.01 (**), 0.001 (***).

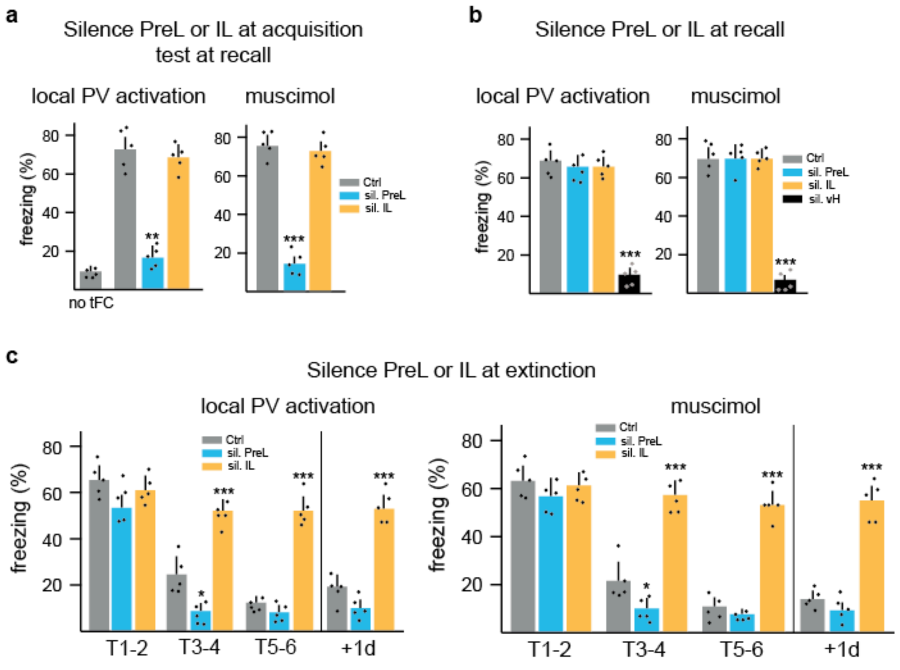

Mukherjee and Caroni Suppl. Fig. 3

Supplementary Figure 3: **Local PreL or IL silencing through PV-neuron activation or muscimol: comparison of behavioral impact.**
a-c: Silencing PreL or IL during acquisition (**a**), recall (**b**) or extinction (**c**) of tFC. Silencing through local pharmacogenetic PV-neuron activation or muscimol had undistinguishable effects: silencing PreL at acquisition specifically impairs freezing at 24h recall (**a**; n=5 each; one-way ANOVA Muscimol: F (2, 12) = 21.85, ***P; Dunnet's *post hoc);* neither PreL nor IL are involved in recall of tFC (**b**; n=5 each; one-way ANOVA: Muscimol F (3, 16) = 29.83, ***P; Dunnet's *post hoc*); silencing IL specifically interferes with extinction learning (**c**; n=5 each; repeat measure two-way ANOVA: Muscimol, F (2, 8) = 61.83, ***P. Error bars: SEM; P<0.05 (*), 0.001 (**), 0001 (***).

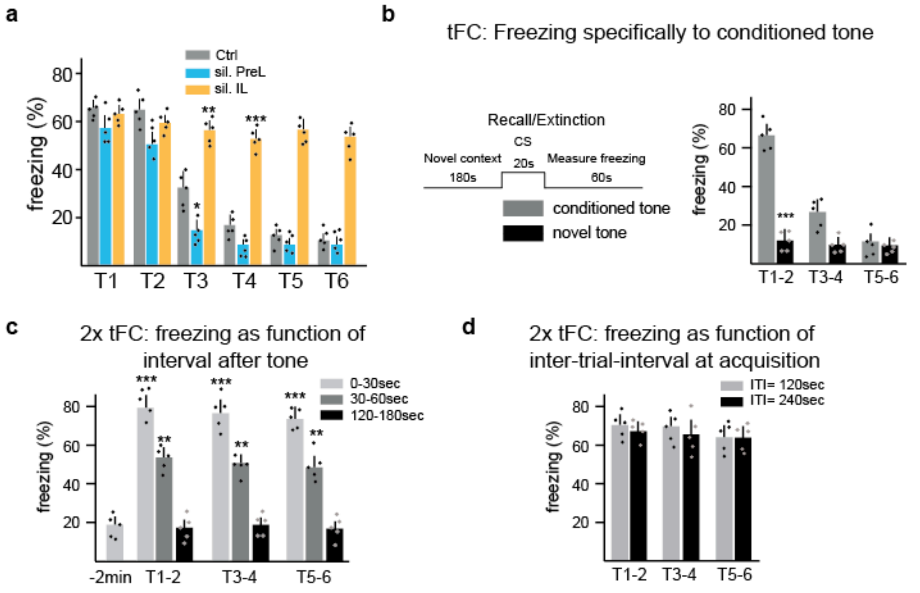

Mukherjee and Caroni Suppl. Fig. 4

<u>Supplementary Figure 4</u>. **Validation of tFC/extinction protocol.**
a: Individual T1-T6 freezing values for extinction experiment as shown in **Fig. 4e**. n = 5 each; Repeat measure two-way ANOVA: Sil. PreL vs. IL F (2, 8) = 96.57, ***P
b: Upon tFC, mice do not freeze to a novel (unconditioned) tone. Recall/extinction protocol. n = 5 each; Repeat measure two-way ANOVA: F (1, 4) = 204.33, ***P
c, d: Freezing as a function of time interval after the tone at recall (**c**), or as a function of inter-trial-interval at acquisition (**d**). Extinction protocol upon 2x tFC (Ext1, no extinction; see **Fig. 5a**). Freezing is highest during the first 30sec after the tone, and absent 120-180sec after the tone (**c**, n = 5 each; Repeat measure two-way ANOVA: F (2, 8) = 188.62, ***P); longer inter trial-intervals at acquisition (240sec instead of 120sec) do not affect freezing at recall (**d**). Two-way ANOVA followed by Tukey's *post hoc*; p<0.05 (*), 0.01 (**), 0.001 (***)

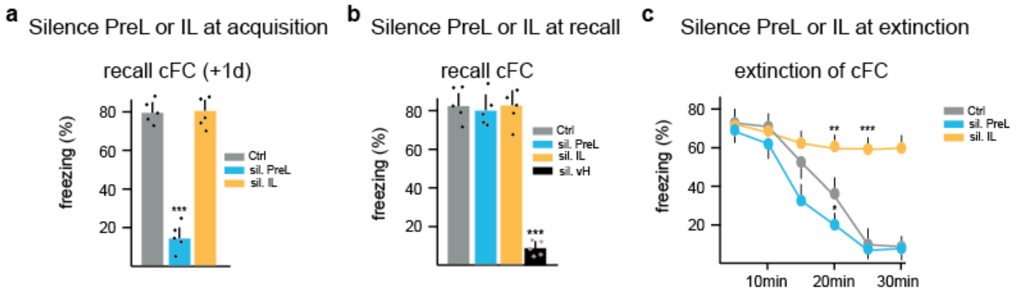

Mukherjee and Caroni Suppl. Fig. 5

<u>Supplementary Figure 5</u>: **PreL promotes contextual fear learning (cFC), IL required for extinction of contextual fear learning.**
Like in tFC, activity in PreL (but not IL) is important at acquisition of cFC for freezing at 24h recall (**a**; n = 5 each; one-way ANOVA: F (2, 12) = 148.9, ***P), neither PreL nor IL is important at recall of cFC (**b**; n = 5 each; one-way ANOVA: F (3, 16) = 97.78, ***P), and activity in IL is required during learning of cFC extinction (**c**; n = 5 each; Repeat measure two-way ANOVA: Sil. PreL vs IL, F (2, 8) = 89.35, ***P). Dunnet's *post hoc* (Tukey's *post hoc* in **c**); p<0.05 (*), 0.01 (**), 0.001 (***).

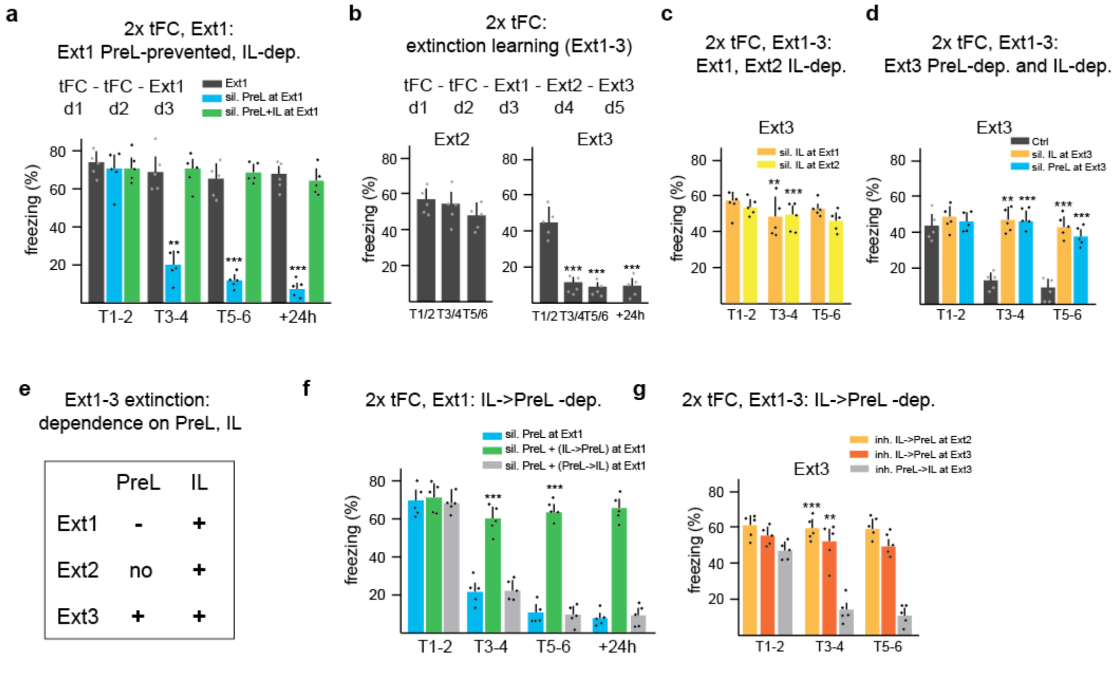

Mukherjee and Caroni Supplementary Fig.6

Supplementary Figure 6. **From IL-dependent to IL- and PreL-dependent extinction learning.**

a: tFC protocols on two consecutive days (2x tFC) lead to PreL-prevented, IL-dependent extinction learning (Ext1). In Ext1, extinction learning is prevented by PreL (blue, silencing PreL), and dependent on IL (green, PreL and IL silenced) (n = 5 each; repeat measure two-way ANOVA: Silence PreL vs PreL+IL, $F(2, 8) = 33.28$, ***P).

b: Ext1-3 learning upon 2x tFC protocol (n = 5 each; one-way ANOVA: Ext3, $F(3, 16) = 25.82$, ***P).

c: Silencing of IL at Ext1 or Ext2 learning suppresses detectable extinction learning at Ext3 (n = 5 each; repeat measure two-way ANOVA: Sil. At Ext1 vs. Ext2, $F(2, 8) = 17.47$, *P).

d: Detectable extinction learning at Ext3 depends on both, IL and PreL (n = 5 each; repeat measure two-way ANOVA: Silence. IL vs. PreL, $F(2, 8) = 13.55$, *P).

e: Summary of PreL and IL roles in Ext1-3 extinction learning. IL is required during Ext1, Ext2, and Ext3 learning. Activity in PreL prevents extinction learning at Ext1; silencing PreL has no detectable effect during Ext2 learning, but it suppresses extinction learning at Ext3.

f: Silencing IL->PreL, but not PreL->IL projection neurons during Ext1 learning suppresses facilitation of PreL silencing, during Ext1 learning (n = 5 each; repeat measure two-way ANOVA: Inh. IL->PreL vs. PreL->IL, $F(2, 8) = 49.87$, ***P).

g: Silencing IL->PreL, but not PreL->IL projection neurons during Ext2 or Ext3 learning reproduces the effect of IL silencing on Ext3 learning (n = 5 each; repeat measure two-way ANOVA: $F(2, 8) = 35.23$, ***P).

Error bars: SEM. Tukey's *post hoc* (Dunnet's *post hoc* in **b, c**); $p<0.05$ (*), 0.001 (**), 0.0001 (***).

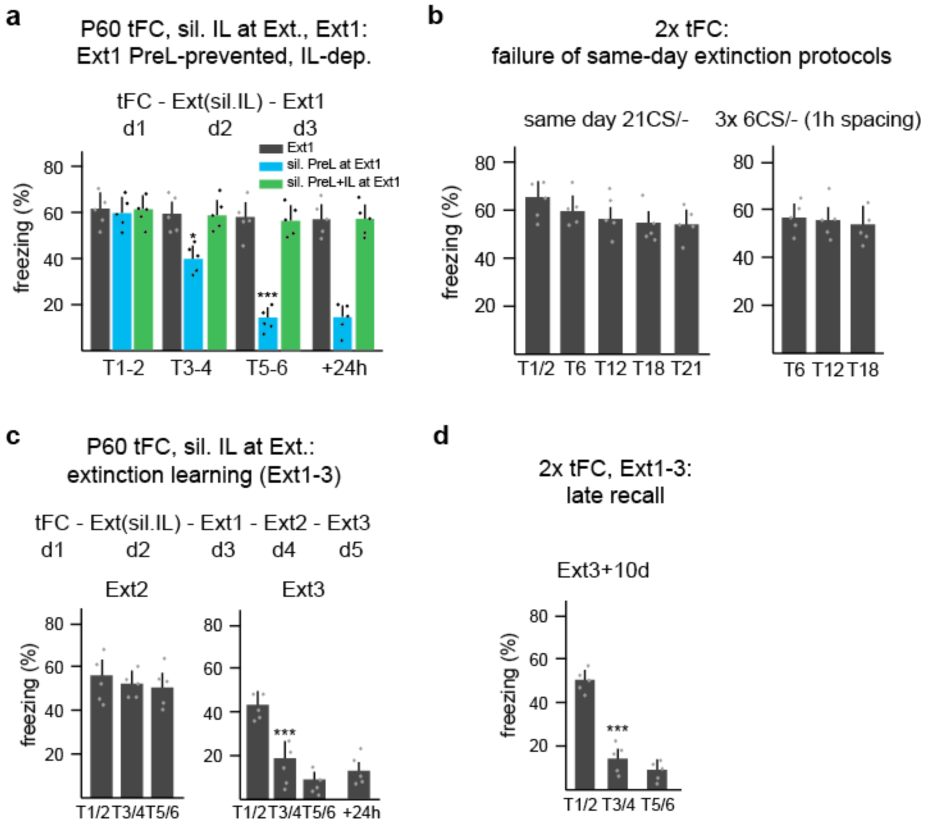

Mukherjee and Caroni Suppl. Fig. 7

Supplementary Figure 7: **Extinction protocols in extinction-resistant mice**.

a: Silencing IL during extinction learning produces PreL-prevented, IL-dependent extinction (Ext1). n = 5 each; Repeat measure two-way ANOVA: $F (2, 8) = 27.38$, ***P.

b: Extinction learning protocols in mice that underwent 2x tFC. 21 CS/- (left) or 3x 6CS/- (center) delivered on the same day fail to produce detectable extinction learning.

c: Ext1-3 learning upon tFC followed by IL silencing during extinction protocol as in (**a**). Right; n = 5 each; one-way ANOVA: $F (3, 16) = 46.87$, ***P.

d: Savings upon Ext1-3 learning. Mice that underwent 2x tFC followed by an Ext1-3 protocol rapidly learn to extinguish again upon return of fear (10d). n = 5 each; one-way ANOVA: $F (2, 12) = 77.77$, ***P. Dunnet's *post hoc* (Tukey's *post hoc* in **a**); p<0.05 (*), 0.01 (**), 0.001 (***).

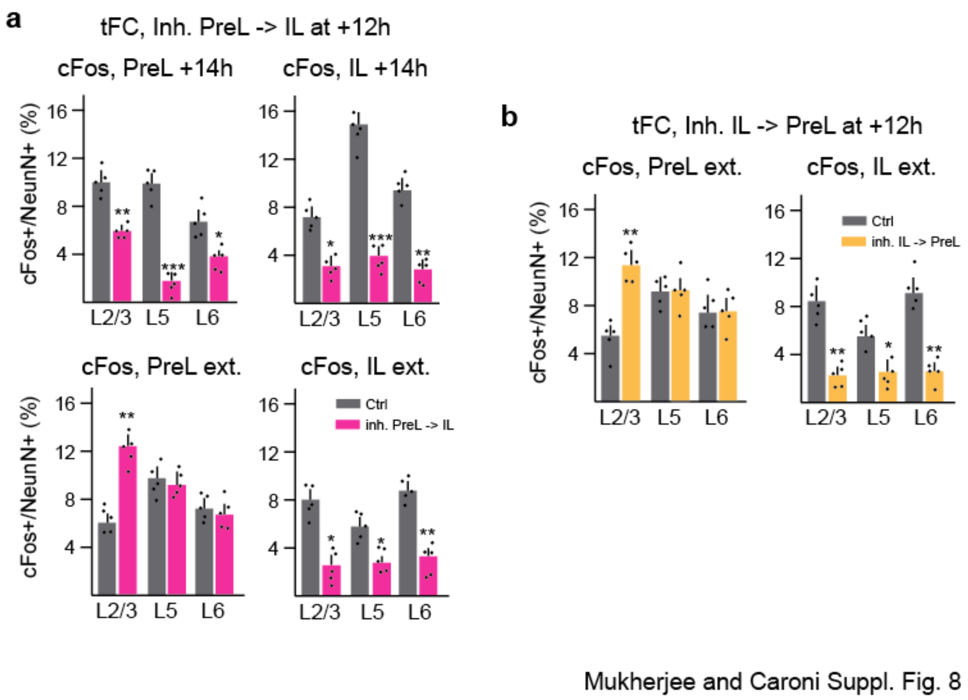

Supplementary Figure 8: **Activity in PreL->IL and in IL->PreL projecting neurons required at +12h-14h after fear learning acquisition to set up role of IL in extinction learning.**

a: Impact of PreL->IL projection neuron silencing at +12h after acquisition on induction of cFos in PreL and IL at +14h after acquisition and upon extinction protocol. n = 5 each; Two-way ANOVA: PreL +14h, F (1, 24) = 38.04, ***P; IL +14h, F (1, 24) = 106, ***P; PreL Ext, F (1, 24) = 5.49, *P; IL Ext, F (1, 24) = 40.57, ***P.

b: Impact of IL->PreL projection neuron silencing at +12h on induction of cFos in PreL and IL upon extinction protocol. n = 5 each; Two-way ANOVA: PreL Ext, F (1, 24) = 11.97, **P; IL Ext, F (1, 24) = 30.68, ***P. Sidak's *post hoc*.

Error bars: SEM; p<0.05 (*), 0.01 (**).

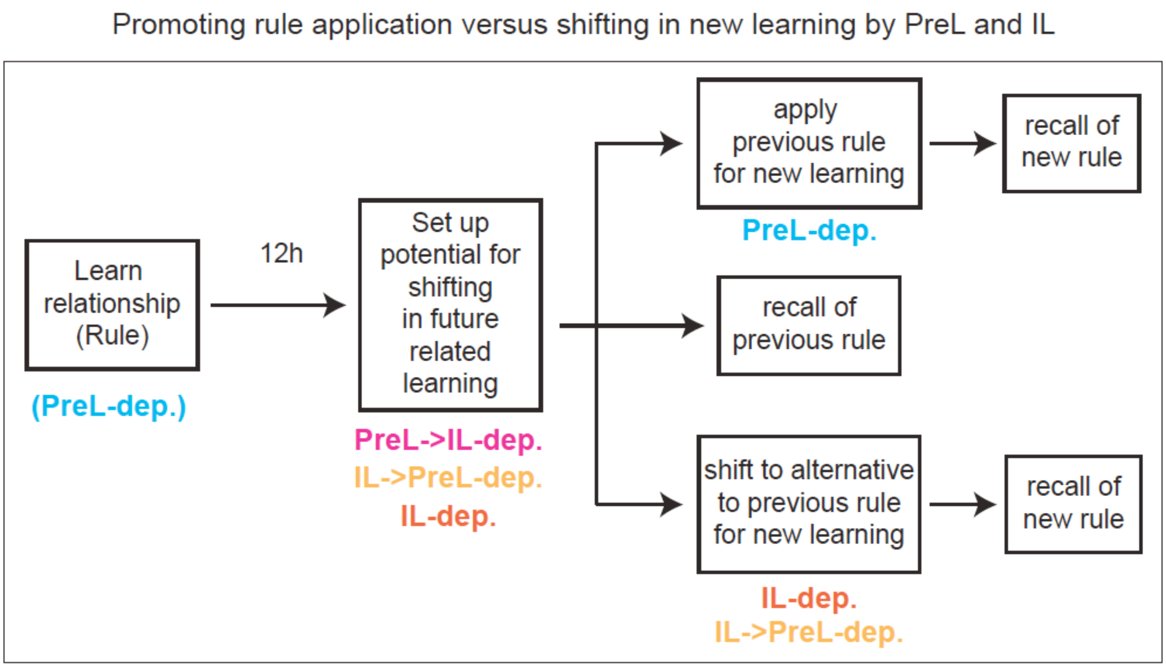

Mukherjee and Caroni, Suppl. Fig. 9

<u>Supplementary Figure 9:</u> Summary of main findings in the study. Requirements for activity in PreL, IL, PreL->IL connectivity, and IL->PreL connectivity during different phases of learning/recall were investigated through pharmacogenetic silencing/inactivation during the learning/recall phase to be tested. The schematic shows how learning through application of a previous rule depends on activity in PreL (cyan), learning requiring shifting to an alternative to the previous rule depends on activity in IL (orange-red) and IL->PreL connectivity (orange), whereas recall of previous learning does not depend on activity in PreL, IL, IL->PreL or PreL->IL connectivity. Activity in IL->PreL and in PreL->IL (magenta) connectivity were both required during a time window approximately 12h after rule learning to set up the role of IL in subsequent alternative learning.
